# Are endometrial cancer clinical practice management guidelines sufficiently consumer centric?

**DOI:** 10.1136/ijgc-2022-003580

**Published:** 2022-04-27

**Authors:** Andreas Obermair, Orla McNally, Rhonda Farrell, Reitan Ribeiro, Joseph Soon-Yau Ng

**Affiliations:** 1 Queensland Centre for Gynaecological Cancer Research, The University of Queensland Centre for Clinical Research, Herston, Queensland, Australia; 2 Department of Gynaecologic Oncology, The Royal Women's Hospital, Parkville, Victoria, Australia; 3 Department of Gynaecology and Cancer Services, University of Melbourne, Parkville, Victoria, Australia; 4 Department of Gynaecologic Oncology, Chris O'Brien Lifehouse, Camperdown, New South Wales, Australia; 5 Department of Gynaecologic Oncology, Royal Prince Alfred Hospital, Camperdown, New South Wales, Australia; 6 Department of Surgery, Erasto Gaertner Hospital, Curitiba, Paraná, Brazil; 7 Department of Surgery, Pontificia Universidade Catolica do Parana, Curitiba, Paraná, Brazil; 8 Department of Obstetrics and Gynecology, National University of Singapore, Singapore; 9 Department of Gynecologic Oncology, National University Cancer Institute, Singapore

**Keywords:** Endometrium, Sentinel Lymph Node, Lymph Nodes

Clinical practice management guidelines for early-stage endometrial cancer suggest surgical staging, including histological assessment of lymph nodes.^1^ Unfortunately, these recommendations are not supported by level 1, randomized evidence on the effectiveness but on evidence from cohort studies (level 2 evidence). If guidelines were consumer centric, they would acknowledge the paucity of high-level evidence and allow for alternative treatments, at least for some patient groups.

Surgical staging is a surgical credo that—despite lack of randomized evidence—has remained standard practice for more than three decades. Since its adoption in 1988 by the International Federation of Gynecology and Obstetrics (FIGO), surgical practice has transitioned into sentinel node biopsy using indocyanine green and near-infrared imaging.^2^ Although high-level evidence is available to suggest that sentinel node biopsy has high diagnostic accuracy,^3^ the evidence for surgical staging is poor when patient-centered outcomes are considered.^4^ Clinical management guidelines currently do not offer patients an informed choice between a hysterectomy with or without lymph node dissection.

## Why patients need a definitive sentinel node biopsy trial in endometrial cancer?

The international ENDO-3 trial aims to deal with this knowledge gap. It randomizes patients to hysterectomy with or without sentinel node biopsy.^5^ The main trial outcome is disease-free survival, complemented by important short-term, patient-centered outcomes.

Some patients voice strong opinions for or against surgical staging through sentinel node biopsy even before specific information is provided. Some have a strong preference for lymph node removal (*“I want peace of mind”, “I want all my cancer removed”*) and fear that not removing lymph nodes puts them at risk. Others voice equally strong concerns against it (*“I want my lymphatic system intact to enjoy excellent general health*”; *“I am scared I will develop lymphedema”*).

High-level evidence informing patients of the benefits and disadvantages of sentinel node biopsy are not yet available. ENDO-3 will provide patients with the necessary high-level information relating to the advantages and potential detriments of sentinel node biopsy compared with no lymphadenectomy, thus quantifying key risks, including the requirement for full lymphadenectomy; the need for post-operative treatment; operative blood loss and adverse events including lymphedema; and the impact on survival probability.

ENDO-3 will address the knowledge gap that has been present for over 30 years and will be critical to inform both clinicians and patients. It will ensure that the choice of surgical management of endometrial cancer by clinicians and patients is supported by robust evidence leading to optimal health outcomes.
